# Measuring recovery among people who have completed residential rehabilitation: Factor structure and scoring of the substance use recovery evaluator

**DOI:** 10.1111/dar.14004

**Published:** 2025-03-04

**Authors:** Emma L. Hatton, Peter J. Kelly, Raimondo Bruno, Joanne Neale, Briony Larance

**Affiliations:** ^1^ Meaningful Outcomes in Substance Use Treatment Centre of Research Excellence & School of Psychology University of Wollongong Wollongong Australia; ^2^ National Drug and Alcohol Research Centre University of New South Wales Sydney Australia; ^3^ School of Psychological Sciences University of Tasmania Hobart Australia; ^4^ National Addiction Centre, Institute of Psychiatry, Psychology & Neuroscience King's College London UK

**Keywords:** factor structure, item scoring, recovery, residential rehabilitation, substance use

## Abstract

**Introduction:**

The substance use recovery evaluator (SURE) is a new patient‐reported outcome measure of recovery from alcohol and other drugs. The original SURE validation study did not include clients from residential rehabilitation treatment, and the possible challenges in applying the measure in this setting were noted. This study evaluates the factor structure and scoring of the substance use recovery evaluator for people after discharge from residential alcohol and other drug rehabilitation in Australia.

**Methods:**

Two hundred and twenty‐five participants interviewed at 14 weeks post‐discharge from residential rehabilitation between 2018 and 2020 were included in a cross‐sectional analysis of longitudinal data. Item response theory statistics (IRT) were used to determine optimal scoring methods for the SURE. Confirmatory factor analysis (CFA) models were used to confirm the SURE's factor structure.

**Results:**

An initial CFA of the 5‐factor model using original scoring could not be fitted. Although IRT indicated a combination of binary and three‐point scale scoring could be used, a binary scale included most of the information from other response categories, and CFA using a Bayes estimation to confirm the original structure with binary data indicated good model fit, *p* = 0.164.

**Discussion and Conclusions:**

The SURE has the same five underlying factors identified by the original study, each of which provides important clinical information about recovery. Binary rescoring provides a valid, parsimonious and clinically relevant way of measuring substance use recovery for residential treatment populations post‐discharge.

**Key points**

- Using item response theory analysis for rescoring.
- Substance use recovery evaluator assessing recovery post‐residential treatment.
- Scoring and factor structure of the substance use recovery evaluator.
- Validating the substance use recovery evaluator for residential treatment attendees.

## 1 INTRODUCTION

Holistic and person‐centred conceptualisations of substance use disorders (SUD) and recovery [1, 2, 3] include both reductions in substance use and improvements in social, occupational and mental health functioning [4, 5]. Movement towards recovery‐oriented and person‐centred practice in the alcohol and other drug (AOD) sector [6, 7, 8] has prompted increasing demand for recovery outcome measures [9, 10]. It is important that people with lived experience are included in measure development to ensure assessments reflect their treatment experiences and validate this population as stakeholders within their own treatment.

Patient‐reported outcome measures (PROM) are questionnaires and rating scales that assess a client's perceived view of their health status, developed through interviews, literature and theory to capture clients' perspectives of their treatment outcome [11, 12]. PROMs can aid clients to engage in self‐reflection and track their own recovery journeys [13]. They can also be used by service providers to inform clinical care [14, 15, 16]. The substance use recovery evaluator (SURE) is a PROM used to evaluate recovery from SUDs that was developed with contributions from service providers and clients from a variety of services, including residential rehabilitation (RR; [10, 17, 18, 19]). The inclusion of this perspective resulted in the first validated measure of recovery, with construct validity and face validity for the population of interest, different from other substance use related outcome measures through the emphasis on patient perspective through the development stage [20]. The wording, appropriateness, utility, importance of all items, and the content and layout of the SURE were evaluated by clients. Factor analysis revealed five underlying subscales: ‘Drinking and Drug Use’, ‘Self‐Care’, ‘Relationships’, ‘Material Resources’ and ‘Outlook on Life’, which correlated positively with existing recovery‐oriented outcome measures, quality of life (WHOQOL‐BREF; [21]) and recovery capital (Assessment of Recovery Capital; [10, 17, 18, 19, 22]).

Ongoing development and application of the SURE has included validating in other languages [23]; however, exploration is still needed in some populations. When publishing the measure, the developers noted that certain SURE items, for example ‘stable housing’ and ‘managing money’ from the Material Resources subscale, may be difficult to complete during a residential rehabilitation episode due to the structure, rules and routines of residential services which limit autonomy and control over these behaviours [20]. Residential treatment is often provided for people with greater dependence severity or other problems, including homelessness or co‐occurring mental illness [24, 25], for which an inpatient setting may provide benefits. People discharged are more likely to be abstinent and have high recovery [2], meaning discharged residential clients are a distinct population from a community sample or people entering RR. There is also an increased emphasis on case management in RR, particularly in discharge planning [26]. Clients may benefit from the structure and routine of residential programs and improvements in nutrition, as well as the assertive linkage with housing, government benefits and other social supports upon discharge [26, 27]. Validation of the SURE has not examined how the measure performs among people discharged from RR.

Valid and reliable PROMs are needed to inform treatment outcomes and quality improvement studies in the residential rehabilitation context. The overarching aims of this study were to undertake a secondary validation study of the SURE in a sample of people discharged from RR, specifically to:

1. Explore the scoring methods for the items and item characteristics of the SURE for people discharged from residential rehabilitation using item response theory.
2. Apply proposed scoring methods suggested in item response theory analyses to compare scoring methods in three confirmatory factor analysis models.
3. Confirm the factor structure of the SURE in populations discharged from residential rehabilitation using confirmatory factor analysis.

This information is needed to provide guidance on the use of the SURE to measure recovery among people discharged from RR.

## 2 METHODS

### 2.1 Setting

Participants were recruited from multiple residential AOD treatment services located across New South Wales and the Australian Capital Territory. The 8 sites included were from three services (We Help Ourselves, The Australian Salvation Army and Adele House) that deliver modified therapeutic community approaches using techniques such as group work, individual counselling and attendance at 12‐step meetings.

### 2.2 Participants

All participants were recruited as part of two larger multi‐site trials, collectively termed the Continuing Care Project (ACTRN 12618001231235), which aimed to assess the feasibility and effectiveness of a telephone‐based intervention after residential rehabilitation discharge. For the CCP study protocol (see Kelly et al. [28]), participants were required to have: (i) a SUD; (ii) attended the treatment facility for greater than four weeks; and (iii) telephone access after treatment discharge. Exclusion criteria for the Continuing Care Project were: (i) expressing current suicide risk; or (ii) being unable to be contacted within two weeks after treatment discharge [29].

The original trial collected data at baseline, 14 and 26 weeks post‐discharge. This study utilises demographic and pre‐treatment data collected at baseline and SURE data collected at the 14‐week assessment. The 14‐week assessment was chosen as this timepoint provided sufficient time to assess recovery post‐discharge, allowing the opportunity to have experienced and/or engaged in the individual SURE items relating to recovery. Of the 304 participants eligible for follow‐up after randomisation, 234 (77% of the original sample) completed a 14‐week assessment. Participants were excluded if any SURE items were missing at the 14‐week post‐discharge follow‐up. This was due to incomplete assessments that were ceased during or before the SURE was completed, or when participants declined to respond to specific SURE items. After exclusion for missing data, 225 remaining participants (92 female) aged between 20 and 71 years (*M* = 39.16 years, SD = 10.23), with complete data on the SURE at the 14‐week assessment, were included in the final sample for validation analyses.

### 2.3 Measures

#### 2.3.1 Demographic and clinical characteristics

Demographic and clinical information collected from participants included age, gender, self‐reported lifetime co‐morbid mental health diagnoses, primary substance of concern and time in treatment.

#### 2.3.2 Substance use recovery evaluator (SURE)

The SURE is a 21‐item PROM assessing recovery from substance use over the previous week with higher scores reflecting greater recovery. Respondents rate each of the 21 items on one of two five‐point scales. Items 1–3 are scored: ‘Every day’ = 1 to ‘Never’ = 5 and items 4–21 are scored ‘None of the time’ = 1 to ‘All of the time’ = 5, with raw total scores ranging from 21 to 105. The developers recommended raw response values be scored between 1 and 3 as follows: for items 1–3: ‘Never’ = 3, ‘On 1 or 2 days’ = 3, ‘On 3 or 4 days’ = 2, ‘On 5 or 6 days’ = 1, and ‘Every day’ = 1 and for items 4–21: ‘All of the time’ = 3, ‘Most of the time’ = 3, ‘A fair amount of the time’ = 2, ‘A little of the time’ = 1 and ‘None of the time’ = 1. Original scores as recommended by the developers therefore ranged from 21 to 63. Item wording and response options for all items are listed in Table 2.

### 2.4 Procedure

Demographic and pre‐treatment information was obtained from participants during a 60‐min baseline assessment conducted by Continuing Care clinicians from the service, in person, prior to residential rehabilitation program completion. The 14‐week assessments were conducted by assessment officers from the research team who were blind to the treatment condition. Proactive follow‐up methods were used including contact by telephone, email, text message, mail or social media (i.e., Facebook). Follow‐up began in June 2018 and was completed in July 2020.

Each 14‐week assessment took approximately 30–40 min to complete. Data were collected and stored in a Research Electronic Data Capture (REDCap) database, hosted by Hunter Medical Research Institute. Participants were reimbursed with 40.00 AUD vouchers, sent via post, for each follow‐up assessment completed. This study was reviewed and approved by the University of Wollongong Human Research Ethics Committee (HE 2018/156).

### 2.5 Statistical analyses

SURE scores breached the assumption of normality with skew towards greater recovery, so predominantly non‐parametric analyses were performed. Descriptive statistics were explored using the Statistical Package for the Social Sciences (SPSS; Version 25) [30].

#### 2.5.1 Identifying optimal scoring methods

Item Response Theory (IRT) item information and item characteristic curves in conjunction with response distributions were used to confirm item inclusion and evaluate scoring. Item information curves are a derivative of the item characteristic curve and display the amount of ‘information’ an individual item provides, with higher levels of information resulting in more accurate score estimates of the latent trait ability [31, 32]. Lower peaks and slopes demonstrate less information. Wavy curves may indicate that information is provided across multiple response categories which are then combined into one waveform for the information curve. Item characteristic curves show distributions for each response category per item and curves depict the discriminative ability of items to differentiate between levels of the trait and a particular response in terms of location on the axes [32]. For each of these figures, scoring can be inferred by the number of overlapping curves, threshold proximities, and response frequencies in individual response curves for each item. Curves which overlap or have proximal thresholds may indicate similar likelihoods of endorsing multiple items. Items with low to no responses in one response category may indicate that fewer response categories or rescoring are needed. In this study, Mplus (Version 8.5) was used to conduct a Graded Response Model IRT using theta parametrisation for the subscales with more than three items (Drinking and Drug Use, Self‐Care and Relationships). A minimum of three items is needed for model identification, similar to confirmatory factor analysis (CFA), so no IRT analyses were conducted for Material Resources or Outlook on Life subscales [33].

#### 2.5.2 Confirming the five‐factor structure of the SURE using multiple scoring methods

Next, the original five‐factor structure suggested in Neale et al. [20] was compared and confirmed by conducting CFA using multiple scoring methods informed by IRT analyses. Two types of model estimation were applied: (i) weighted least squares estimation with mean and variance adjustment; and (ii) Bayesian estimation with normal priors fixed to 1 for latent factor loadings and variances were used to account for positive definiteness and correct for Heywood cases. All CFA models were conducted in Mplus (Version 8.5) and were conducted for all items in the SURE [34]. Model fit statistics, tetrachoric correlations and response distributions were used to assess the appropriateness of each model regarding the factor structure, scoring and whether items within each factor still fit appropriately together for the current study's sample. Items with low variation across categories and poor fit or low inter‐item correlations within factors were assessed for inclusion or exclusion from the final model. Model fit statistics assessed were relative chi‐square (values closer to 2 indicate better model fit [35]), root mean square error of approximation (RMSEA), standardised root mean squared residuals (SRMR; for RMSEA and SRMR values under 0.8 indicate adequate model fit [36, 37]), comparative fit indices (CFI) and Tucker–Lewis indices (TLI; for CFI and TLI values higher than 0.9 are required for close model fit [38, 39]). Model fit for Bayesian models was assessed using 95% confidence interval for chi‐square and *p* values, where *p* values over 0.05 indicated good model fit.

Item characteristic curves were examined for all items. A spread of scores was needed to ensure items adequately captured the sample's recovery, and the raw original and proposed scoring methods were applied. A total of nine models were tested and compared:

- Model I (5 factors, raw 5‐point scale, weighted least squares estimation): CFA was conducted on the raw five‐point scoring for all SURE items (1,2,3,4,5).
- Model II (5 factors, original 3‐point scale, weighted least squares estimation): CFA was conducted on the three‐point scoring method recommended by the original SURE authors [20] who evaluated scoring using IRT item characteristic curves, which indicated that the two highest and two lowest categories could be merged when scoring without losing relevant clinical information. It was recommended that SURE data be restructured and recoded from a five‐point to a three‐point scale. This was done by recoding original scores of one or two to a new score of one, recoding original scores of three to a new score of two, and recoding original scores of four or five to a new score of three [20], so that scores range from 21 to 63, with higher scores indicating greater recovery (1–2,3,4–5).
- Model III (5 factors, original 3‐point scale, Bayesian estimation): CFA was conducted on the original three‐point scoring method using Bayesian estimation to account for any Heywood cases (1–2,3,4–5)
- Model IV (5 factors, revised mixed 2‐ or 3‐point scale, weighted least squares estimation): CFA was conducted on the revised mixed two‐ and three‐point scoring methods recommended by IRT item characteristic curves (1–4,5, or, 1–3,4,5).
- Model V (5 factors, revised 3‐point scale, weighted least squares estimation): CFA was conducted using the revised three‐point scoring method recommended by IRT item characteristic curves for all items (1–3,4,5).
- Model VI (5 factors, revised binary scale, weighted least squares estimation): CFA was conducted using the revised binary scoring method recommended by IRT item characteristic curves for all items (1–4,5).
- Model VII (5 factors, revised mixed 2‐ or 3‐point scale, Bayesian estimation): CFA was conducted on the revised mixed two‐ and three‐point scoring methods recommended by IRT item characteristic curves, applying Bayesian estimation to account for any Heywood cases (1–4,5, or, 1‐3,4,5).
- Model VIII (5 factors, revised 3‐point scale, Bayesian estimation): CFA was conducted using the revised three‐point scoring method recommended by IRT item characteristic curves for all items, applying Bayesian estimation to account for any Heywood cases (1–3,4,5).
- Model IX (5 factors, revised 2‐point scale, Bayesian estimation): CFA was conducted using the revised binary scoring method recommended by IRT item characteristic curves for all items, applying Bayesian estimation to account for any Heywood cases (1–4,5).

## 3 RESULTS

### 3.1 Sample characteristics

The sample was predominantly male (59%) with an average age of 39.2 years (Table 1). Most participants (80%) reported receiving benefits, allowances or pensions as a main source of income in the previous year. Amphetamine‐type stimulants (59.1%) and alcohol (56.9%) were the most common substances of concern. However, it was common for participants to report polysubstance use, with 69% having at least two primary substances of concern and 35% having at least three primary substances of concern. The most commonly reported combinations of primary substances of concern were: amphetamine + cannabis (17.3%), amphetamines + alcohol (13.1%), alcohol + cannabis (11.5%) and alcohol + amphetamines + cannabis (10.2%). Mental health service utilisation and prior mental health diagnoses were common, with 74.7% of participants reporting prior treatment and 72.4% reporting a previous diagnosis of a mental health disorder.

**TABLE 1:** Demographic characteristics of participants at baseline (*N* = 225).

	*n*/*M* ^e^	%/SD^e^
Demographic characteristics		
Gender		
Male	132	58.7
Female	92	40.9
Other	1	0.4
Age, years	39.16^e^	10.23^e^
Time in treatment, weeks	11.96^e^	8.80^e^
Country of birth		
Australia	202	89.8
Other/unknown	23	11.2
Main source of income in previous year^a^, ^b^		
Benefit/allowance/pension	180	80.0
Wage/salary	70	31.1
Other	9	4.0
Own business	8	3.6
No income	5	2.2
Superannuation	4	1.8
Worker's compensation/accident or sickness insurance	1	0.4
Education		
Year 10	77	34.2
Tertiary	71	31.6
Year 12	39	17.3
Year 7–9	28	12.4
Other/unknown	10	4.4
Marital status		
Single, never married	144	64.0
Divorced/separated	54	24.0
Married/de facto	23	10.2
Widowed	3	1.3
Other/unknown	1	0.4
Mental health diagnosis^a^, ^c^		
Mania or bipolar	36	16.0
Schizophrenia	14	6.2
Drug‐induced psychosis	39	17.3
Other psychosis	10	4.4
Depression	135	60.0
Anxiety	117	52.0
AD/HD	28	12.4
PTSD	53	23.6
Personality disorder	20	8.9
Other/unknown	1	0.4
Substance use		
Primary substance of use^a^, ^d^		
Amphetamine type substances	133	59.1
Alcohol	128	56.9
Cannabis	86	38.2
Benzodiazepines	29	12.9
Heroin	26	11.5
Other opioids	24	10.6
Cocaine	19	8.4
Other/unknown	14	6.2

^a^ Percentage of the total.

^b^ Some participants reported having more than one main source of income.

^c^ Some participants reported having more than one previously diagnosed mental health disorder.

^d^ Some participants reported having more than one primary substance of use.

^e^ Denotes mean or standard deviation.

Abbreviations: AD/HD, attention‐deficit/hyperactivity disorder; PTSD, post traumatic stress disorder.

### 3.2 Item response distribution and information

All items were skewed indicating higher levels of recovery (see Table 2). Items 1 (‘I have drunk too much’) and 2 (‘I have used street drugs’) had very few responses in response categories 1 (‘none of the time'/‘every day’), 2 (‘a little of the time'/‘on 5 or 6 days’), and 3 (‘a fair amount of the time'/‘on 3 or 4 days’); Item 13 (‘I have felt supported by people around me’) had no responses in response categories 1–3. Distributions indicated that item 20 (‘I have felt positive’) and item 19 (‘I have felt happy with my overall quality of life’) had the highest proportions of higher responses, indicating higher recovery (38.2% and 37.3% ‘all of the time’, respectively). Item 15 (‘I have treated others with respect and consideration’) had the lowest proportion of participants reporting higher responses (13.3% ‘all of the time’), indicating lower recovery. Total scores ranged from 53 to 104 (median = 88, interquartile range = 7) with the original five‐point scoring, and from 36 to 63 (median = 63, interquartile range = 0) with the original three‐point (1–5) scoring.

**TABLE 2:** Item wording, response distributions, mean raw reversed item scores and optimal scoring of individual substance use recovery evaluator items (*N* = 225).

								Proposed scoring	
Item wording		Every day (%)	On 5 or 6 days (%)	On 3 or 4 days (%)	On 1 or 2 days (%)	Never (%)	Mean (SD)	Binary scale	Three point scale
1	I have drunk too much	0.0	0.9	0.0	81.3	17.8	4.16 (0.43)	X	
2	I have used street drugs	0.9	0.0	0.0	76.4	22.7	4.20 (0.52)	X	
3	I have experienced cravings	1.3	1.8	0.4	64.9	31.6	4.24 (0.68)	X	

		None of the time (%)	A little of the time (%)	A fair amount of the time (%)	Most of the time (%)	All of the time (%)	Mean (SD)	Binary scale	Three point scale
4	I have coped with problems without misusing drugs or alcohol	0.0	1.3	0.9	71.6	26.2	4.23 (0.52)		X
5	I have managed pains and ill‐health without misusing drugs or alcohol	0.4	0.4	0.4	75.1	23.6	4.21 (0.51)		X
6	I have been spending my free time on hobbies and interests that do not involve drugs or alcohol	1.3	0.9	0.4	70.2	27.1	4.21 (0.62)		X
7	I have been taking care of my mental health	0.4	0.4	2.7	68.9	27.6	4.23 (0.56)	X	
8	I have been taking care of my physical health	0.9	0.4	3.6	56.9	38.2	4.31 (0.65)		X
9	I have been eating a good diet	0.9	0.9	4.0	56.4	37.8	4.29 (0.67)	X	
10	I have slept well	0.9	2.2	3.6	53.8	39.6	4.29 (0.72)	X	
11	I have had a good daily routine	0.9	0.9	3.1	58.2	36.9	4.29 (0.66)		X
12	I have been getting on well with people	0.0	0.4	0.9	78.2	20.4	4.19 (0.44)	X	
13	I have felt supported by people around me	0.0	0.0	0.0	75.1	24.9	4.25 (0.43)	X	
14	I have been treated with respect and consideration by people around me	0.0	0.0	1.3	80.0	18.7	4.17 (0.41)		X
15	I have treated others with respect and consideration	0.0	0.0	0.9	85.8	13.3	4.12 (0.36)	X	
16	I have had stable housing	0.4	0.0	0.4	82.7	16.4	4.15 (0.43)		
17	I have had a regular income (from benefits, work, or other legal sources)	0.0	0.0	1.3	80.4	18.2	4.17 (0.41)		
18	I have been managing my money well	0.0	1.3	3.6	60.9	34.2	4.28 (0.60)		
19	I have felt happy with my overall quality of life	0.4	0.4	1.8	60.0	37.3	4.33 (0.58)		
20	I have felt positive	0.0	0.9	3.1	57.8	38.2	4.33 (0.58)		
21	I have had realistic hopes and goals for myself	0.0	0.9	2.7	65.8	30.7	4.26 (0.55)		

*Note*: For item characteristic curves, see Appendix D (Figures D1, D2, D3, D4, D5, D6) for Drinking and Drug Use Subscale (Items 1–6), Appendix E (Figures E1, E2, E3, E4, E5) for Self‐Care Subscale (Items 7–11), and Appendix F (Figures F1, F2, F3, F4) for Relationships Subscale (Items 12–15). It contains item scores, where higher scores indicate higher recovery, with no additional rescoring or collapsing of categories. The binary scale (1–4, 5) and three‐point scale (1–3, 4, 5) columns refer to the categorisation of recommended scorings for individual items based on the Item Response Theory analyses conducted in the current study.

**FIGURE A1:**
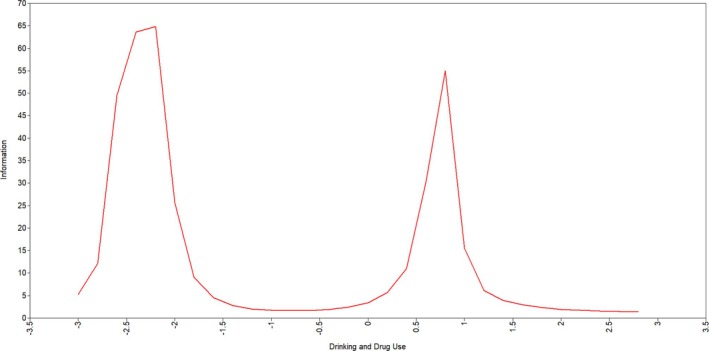
Total information curve for the Drinking and Drug Use Subscale.

**FIGURE A2:**
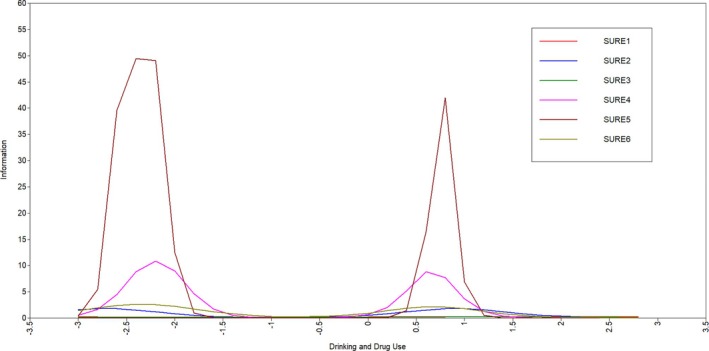
Total information curve for all items in the Drinking and Drug Use Subscale.

**FIGURE B1:**
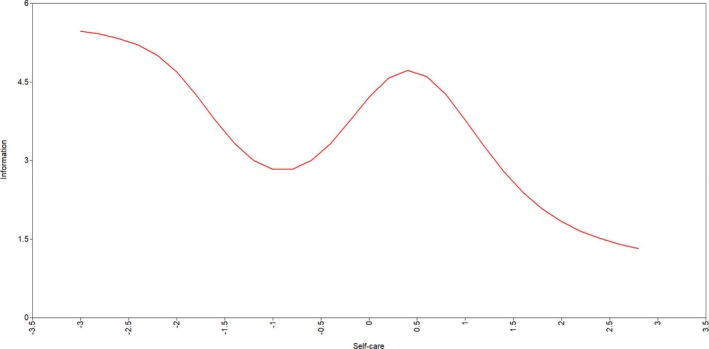
Total information curve for the Self‐Care Subscale.

**FIGURE B2:**
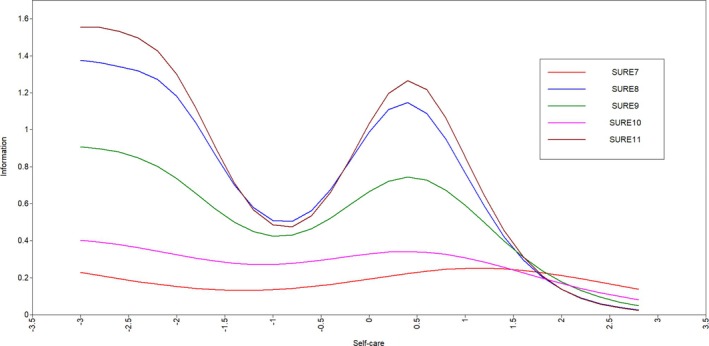
Total information curve for all items in the Self‐Care Subscale.

**FIGURE C1:**
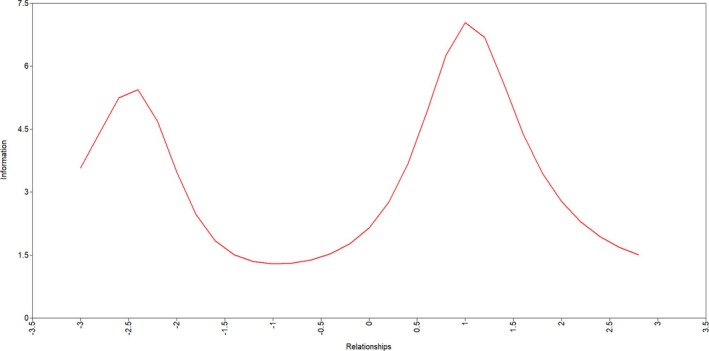
Total information curve for the Relationships Subscale.

**FIGURE C2:**
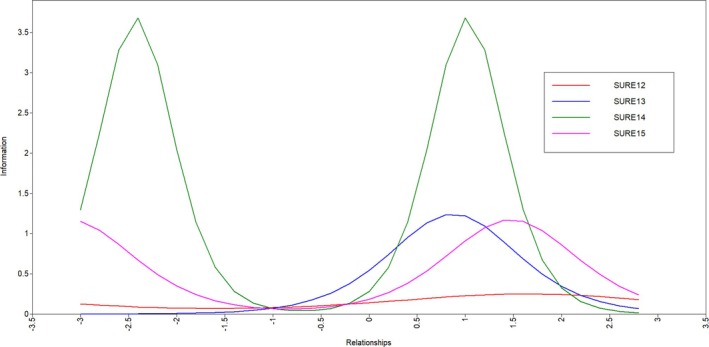
Total information curve for all items in the Relationships Subscale.

Subscale total item information curves and information curves for individual items within the Drinking and Drug Use (Appendix A, Figures A1 and A2), Self‐Care (Appendix B, Figures B1 and B2) and Relationships subscales (Appendix C, Figures C1 and C2) present the amount of information at each level of the latent trait provided by the items within each subscale. The latent trait (subscale) was standardised with a mean of 0. Negative peaks indicated items which were useful for measuring lower levels of recovery for each subscale, while positive peaks indicated items better for determining higher recovery. Items with clear apex points (like items 3, 5 and 14) had higher discrimination or greater precision whereas items with smoother or broader peaks (like items 8, 9 and 11) had lower relative precision but provided information on a broader range of the latent trait. Most information curves had bimodal distributions with two clear peaks, however, curves varied regarding the level of the latent trait and the level of discrimination for each item. Across subscales, item 5 (‘I have managed pains and ill‐health without misusing drugs or alcohol’; e.g., *x* = −2.5, *y* = 50) from the Drinking and Drug Use subscale, item 11 (‘I have had a good daily routine’; e.g., *x* = 0.5, *y* = 1.3) in Self‐Care, and item 14 (‘I have been treated with respect and consideration by people around me’; e.g., *x* = −2.5, *y* = 3.5) in Relationships, provided the greatest information. The relative peaks for Drinking and Drug Use were much greater than other subscales, indicating this scale was more precise at the levels of the latent trait that coincide with those peaks.

### 3.3 Using item characteristic curves to determine optimal scoring

**FIGURE D1:**
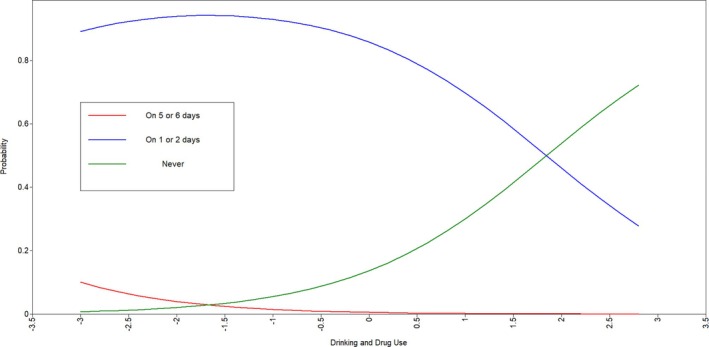
Item characteristic curve for SURE Item 1 (‘I have drunk too much’) in the Drinking and Drug Use Subscale.

**FIGURE D2:**
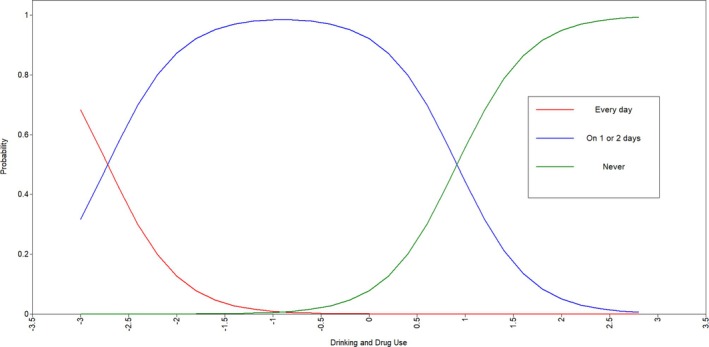
Item characteristic curve for SURE Item 2 (‘I have used street drugs’) in the Drinking and Drug Use Subscale.

**FIGURE D3:**
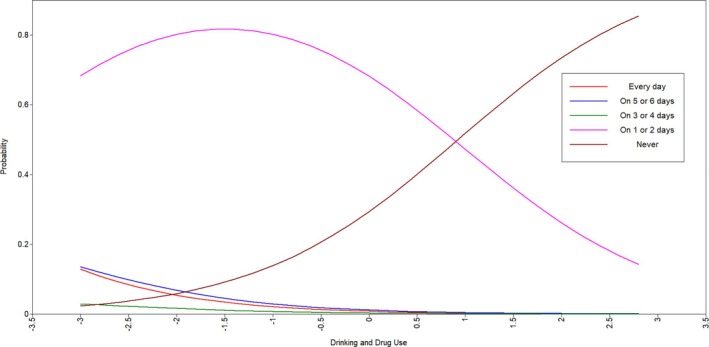
Item characteristic curve for SURE Item 3 (‘I have experienced cravings’) in the Drinking and Drug Use Subscale.

**FIGURE D4:**
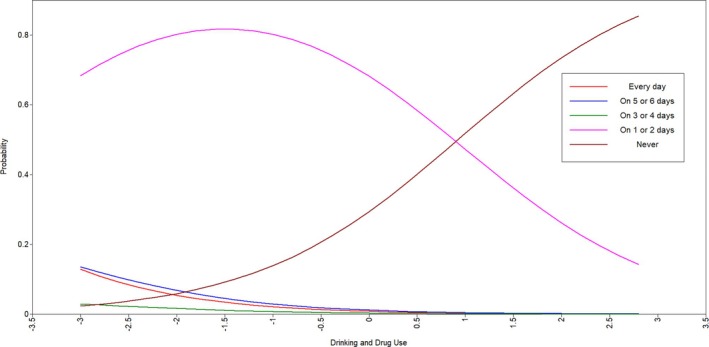
Item characteristic curve for SURE Item 4 (‘I have coped with problems without misusing drugs or alcohol’) in the Drinking and Drug Use Subscale.

**FIGURE D5:**
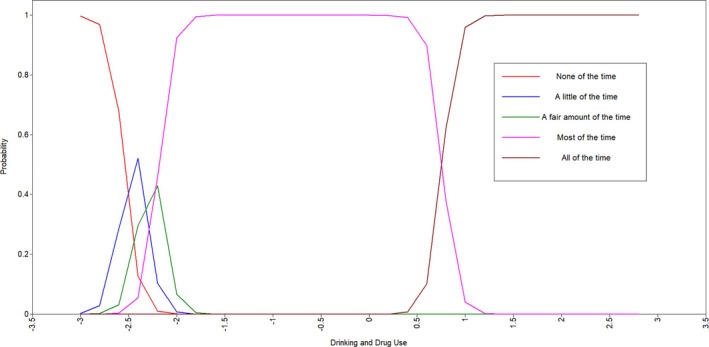
Item characteristic curve for SURE Item 5 (‘I have managed pains and ill‐health without misusing drugs or alcohol’) in the Drinking and Drug Use Subscale.

**FIGURE D6:**
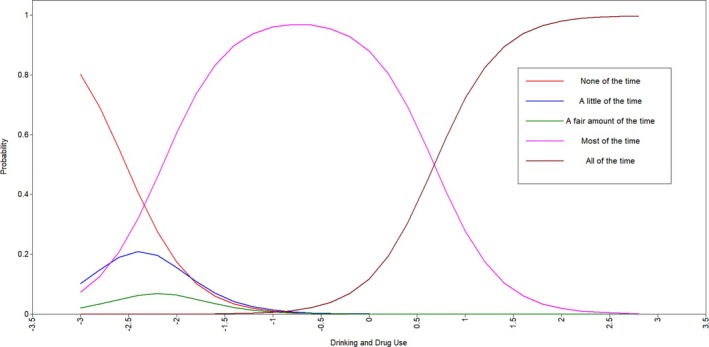
Item characteristic curve for SURE Item 6 (‘I have been spending my free time on hobbies and interests that do not involve drugs and alcohol’) in the Drinking and Drug Use Subscale.

**FIGURE E1:**
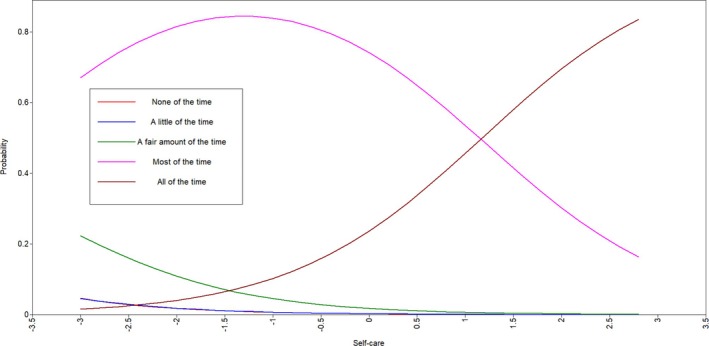
Item characteristic curve for SURE Item 7 (‘I have been taking care of my mental health’) in the Self‐Care Subscale.

**FIGURE E2:**
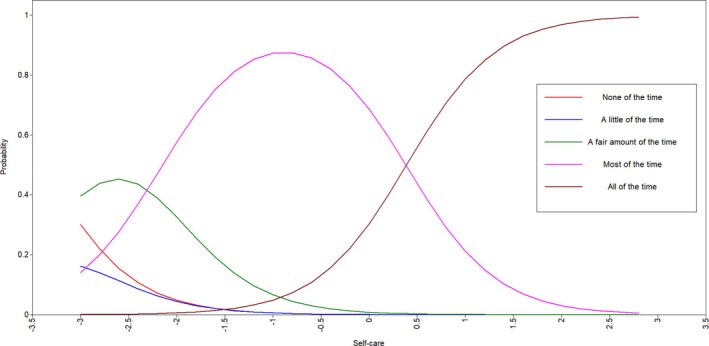
Item characteristic curve for SURE Item 8 (‘I have been taking care of my physical health’) in the Self‐Care Subscale.

**FIGURE E3:**
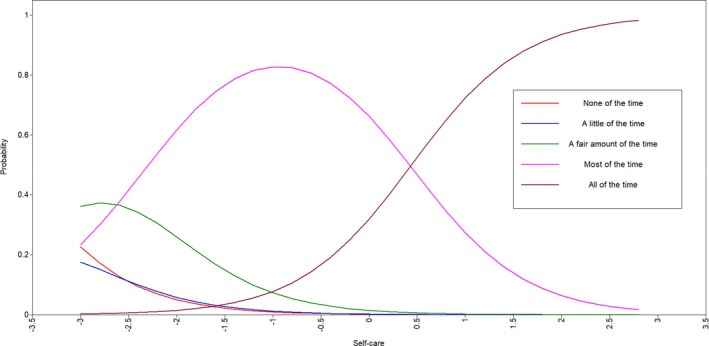
Item characteristic curve for SURE Item 9 (‘I have been eating a good diet’) in the Self‐Care Subscale.

**FIGURE E4:**
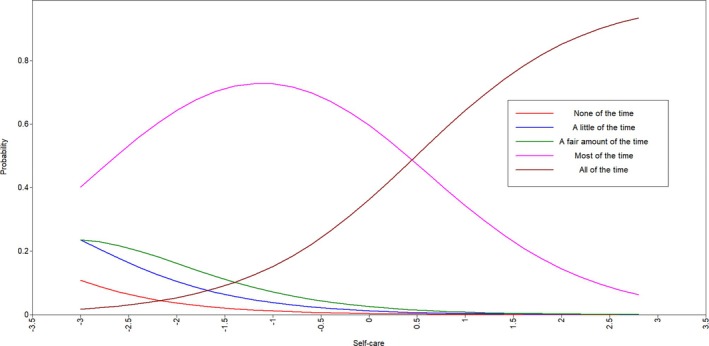
Item characteristic curve for SURE Item 10 (‘I have slept well’) in the Self‐Care Subscale.

**FIGURE E5:**
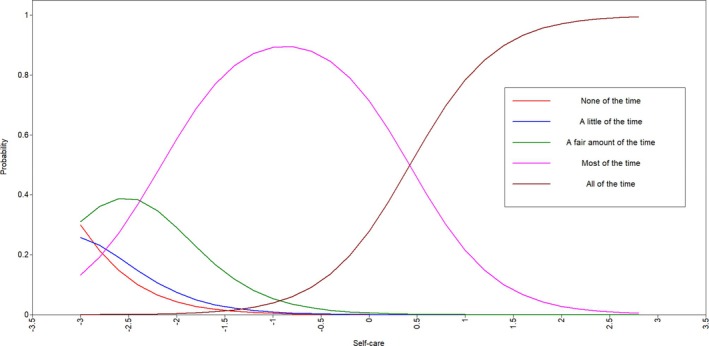
Item characteristic curve for SURE Item 11 (‘I have had a good daily routine’) in the Self‐Care Subscale.

**FIGURE F1:**
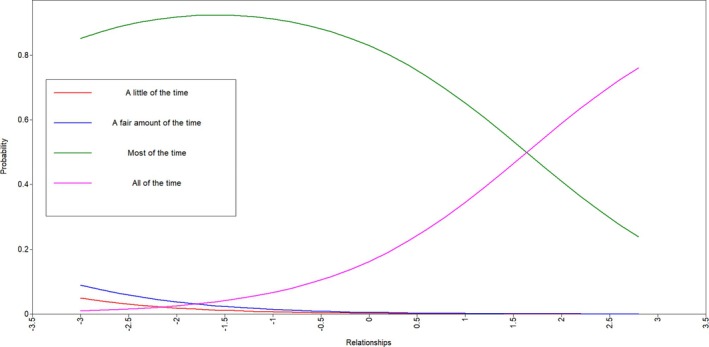
Item characteristic curve for SURE Item 12 (‘I have been getting on well with people’) in the Relationships Subscale.

**FIGURE F2:**
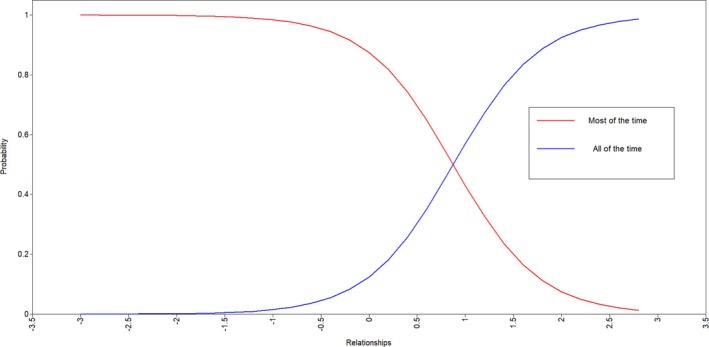
Item characteristic curve for SURE Item 13 (‘I have felt supported by people around me’) in the Relationships Subscale.

**FIGURE F3:**
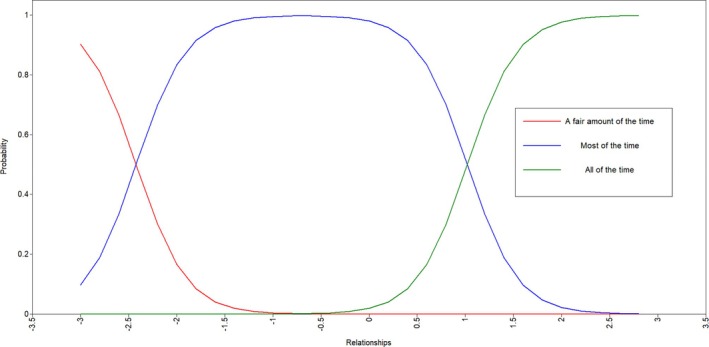
Item characteristic curve for SURE Item 14 (‘I have been treated with respect and consideration by people around me’) in the Relationships Subscale.

**FIGURE F4:**
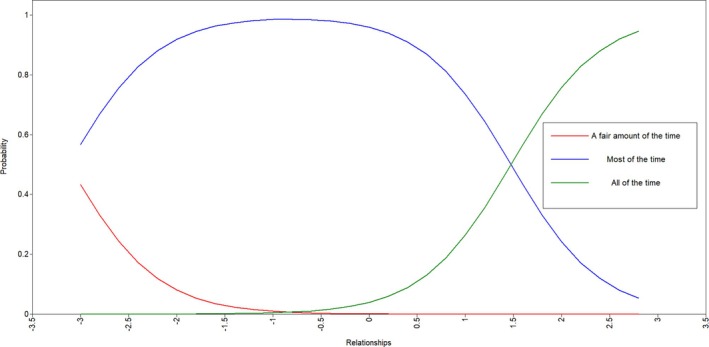
Item characteristic curve for SURE Item 15 (‘I have treated others with respect and consideration’) in the Relationships Subscale.

Item characteristic curves for Drinking and Drug Use (Appendix D, Figures D1, D2, D3, D4, D5, D6), Self‐Care (Appendix E, Figures E1, E2, E3, E4, E5) and Relationships (Appendix F, Figures F1, F2, F3, F4) were evaluated using raw 5‐point scores and suggested three possible scoring methods based on overlapping categories for the individual SURE items and subscales: (i) mixed scoring using a combination of binary and three‐point scoring; (ii) binary (1–5) scoring of all items; and (iii) three‐point (1–5) scoring for all items. Specifically, the data indicated that response categories 1–4 (‘None of the time’ to ‘Most of the time’) could be merged for binary response categories and response categories 1–3 (‘None of the time’ to ‘A fair amount of the time’) could be merged for the three‐point scoring. Groupings of responses into three‐response categories indicated in this study differed from Neale et al. [20] with categories 1–3 merged in this study as opposed to merging 1–2 and 4–5 in the original paper.

The mixed scoring method applied both binary and three‐point scoring dependent on the indications of the item characteristic curves (Table 2). Binary scoring would be appropriate for nine SURE items, and three‐point scoring would be appropriate for six items from the Drinking and Drug Use, Self‐Care and Relationships subscales. As the Material Resources and Outlook on Life subscales could not be tested using IRT analyses due to the small number of items within the subscales (*n* = 3), all items in these subscales were scored with new three‐point scoring as identified by the item characteristic curves for the mixed scoring method to ensure no additional statistical information was lost.

### 3.4 Factor structure

Item response frequencies at the 14‐week assessment indicated insufficient spread of responses to complete CFA (Table 2). Consequently, Model I chi‐square statistic was significant, indicating poor model fit when scored with five response categories (*χ* ^2^ = 917.115, RMSEA = 0.135, CFI = 0.867, TFI = 0.843, SRMR = 0.114). The original five‐factor model with three response categories, as proposed by Neale et al. [20] was attempted with a maximum likelihood approximation (Model II) and Bayesian estimation (Model III), but could not be conducted due to low response variation in lower response categories. Measurement models within subscales indicated adequate model fit for Drinking and Drug Use (*χ* ^2^ = 34.282, RMSEA = 0.112, CFI = 0.991, TFI = 0.985, SRMR = 0.032) and poor model fit for Self‐Care (*χ* ^2^ = 36.847, RMSEA = 0.168, CFI = 0.952, TFI = 0.903, SRMR = 0.046) and Relationships (*χ* ^2^ = 18.691, RMSEA = 0.193, CFI = 0.982, TFI = 0.947, SRMR = 0.041). See Table 3 for CFA model fit comparisons.

**TABLE 3:** Goodness of fit indices for the five‐factor substance use recovery evaluator models (*N* = 225).

Model	Description	*χ* ^2^	95% CI	*p*	RMSEA	CFI	TLI	SRMR
I	5 factors, 5‐point scale	917.155	–	<0.001	0.135	0.867	0.843	0.114
VI	5 factors, binary scale	404.35	–	<0.001	0.075	0.967	0.961	0.121
VII	5 factors, mixed scale, Bayesian estimation	–	[7.18, 156.92]	0.020	–	–	–	–
VIII	5 factors, revised 3‐point scale, Bayesian estimation	–	[26.59, 164.74]	0.007	–	–	–	–
IX	5 factors, binary scale, Bayesian estimation	–	[−32.18, 110.33]	0.164	–	–	–	–

*Note*: Models II–V did not converge and have been excluded from the above. Confirmatory factor analysis for Models II, III and IV could not be conducted. Abbreviations: χ^2^, model chi‐squared; CFI, Comparative Fit Index; CI, confidence interval for the difference between observed and the replicated chi‐square values; RMSEA, Root Mean Square Error of Approximation; SRMR, Standardised Root Mean Square Residual; TLI, Tucker–Lewis Index.

After the IRT analyses, CFA for the five‐factor model was conducted again using mixed scoring (Model IV), three‐point scoring (Model V) and binary scoring (Model VI). CFA could not be conducted with mixed scoring nor three‐point scoring. Model fit did not reach significance for the five‐factor model with binary scoring (*χ* ^2^ = 404.353, RMSEA = 0.075, CFI = 0.967, TFI = 0.961, SRMR = 0.121), nor the single restricted models for any scoring; however, the analyses indicated potential Heywood cases. Heywood cases are related to issues where there may not be ‘positive definiteness’ meaning there may be negative covariances in the variance–covariance matrices [40]. These can be handled using Bayes estimation and using priors to define the covariance parameters.

Using Bayes estimation, the five‐factor model indicated poor model fit for the mixed (Model VII; *p* = 0.020) and three‐point (Model VIII; *p* = 0.007) scoring. Finally, acceptable model fit was indicated for the five‐factor model with binary scoring (Model IX; *p* = 0.164). Total scores ranged from 21 to 41 (*M* = 26.81, SD = 5.19) with the proposed binary (1–4,5) scoring. For Model IX, standardised factor loadings are shown in Table 4 and tetrachoric correlations are shown in Table 5. Tetrachoric correlations range between 0.11 and 0.89 for all SURE items. Item 18 (‘I have been managing my money well’) had the lowest factor loading (0.301) and inter‐item correlations (0.27 and 0.24) within any factor. However, as overall model fit was adequate, and the Material Resources items provide clinically relevant information, this item was retained in the model. Statistical interpretation of the Material Resource subscale should be considered with caution.

**TABLE 4:** Standardised factor estimates for the 21‐item substance use recovery evaluator using Bayes estimation and binary scoring (Model IX; *N* = 225).

	Loadings				
Item	Drinking and drug use	Self‐care	Relationships	Material resources	Outlook on life
1. I have drunk too much	0.867				
2. I have used street drugs	0.920				
3. I have experienced cravings	0.583				
4. I have coped with problems without misusing drugs or alcohol	0.938				
5. I have managed pains and ill‐health without misusing drugs or alcohol	0.949				
6. I have been spending my free time on hobbies and interests that do not involve drugs or alcohol	0.913				
7. I have been taking care of my mental health		0.854			
8. I have been taking care of my physical health		0.707			
9. I have been eating a good diet		0.574			
10. I have slept well		0.475			
11. I have had a good daily routine		0.764			
12. I have been getting on well with people			0.822		
13. I have felt supported by people around me			0.739		
14. I have been treated with respect and consideration by people around me			0.837		
15. I have treated others with respect and consideration			0.901		
16. I have had stable housing				0.897	
17. I have had a regular income (from benefits, work, or other legal sources)				0.812	
18. I have been managing my money well				0.301	
19. I have felt happy with my overall quality of life					0.889
20. I have felt positive					0.917
21. I have had realistic hopes and goals for myself					0.871

*Note*: Cross‐loadings were not explored to reduce model parameters and avoid issues with convergence. As model fit was appropriate for the binary Bayesian model, modification indices and cross‐loadings were not reviewed for this model.

**TABLE 5:** Substance use recovery evaluator inter‐item tetrachoric correlations for the binary Bayesian model (Model IX).

	1	2	3	4	5	6	7	8	9	10	11	12	13	14	15	16	17	18	19	20
1	–																			
2	0.80	‐																		
3	0.51	0.54	‐																	
4	0.81	0.86	0.55	‐																
5	0.82	0.87	0.55	0.89	‐															
6	0.79	0.84	0.53	0.86	0.87	‐‐														
7	0.44	0.47	0.30	0.48	0.49	0.47	‐													
8	0.37	0.39	0.25	0.40	0.40	0.39	0.60	‐												
9	0.30	0.32	0.20	0.32	0.33	0.31	0.49	0.41	‐											
10	0.25	0.26	0.17	0.27	0.27	0.26	0.41	0.34	0.27	‐										
11	0.40	0.42	0.27	0.43	0.43	0.42	0.65	0.54	0.44	0.36	‐									
12	0.48	0.51	0.32	0.52	0.53	0.51	0.49	0.40	0.33	0.27	0.44	‐								
13	0.43	0.46	0.29	0.47	0.47	0.46	0.44	0.36	0.29	0.24	0.39	0.61	‐							
14	0.49	0.52	0.33	0.53	0.54	0.52	0.50	0.41	0.33	0.28	0.44	0.69	0.62	‐						
15	0.53	0.56	0.35	0.57	0.58	0.55	0.53	0.44	0.36	0.30	0.48	0.74	0.67	0.75	‐					
16	0.69	0.73	0.47	0.75	0.76	0.73	0.39	0.32	0.26	0.22	0.35	0.57	0.52	0.58	0.63	‐				
17	0.63	0.66	0.42	0.68	0.69	0.66	0.35	0.29	0.24	0.20	0.32	0.52	0.47	0.53	0.57	0.73	‐			
18	0.23	0.25	0.16	0.25	0.25	0.24	0.13	0.11	0.09	0.07	0.12	0.19	0.17	0.20	0.21	0.27	0.24	‐		
19	0.35	0.37	0.23	0.38	0.38	0.37	0.69	0.57	0.47	0.39	0.62	0.51	0.46	0.52	0.56	0.33	0.30	0.11	‐	
20	0.36	0.38	0.24	0.39	0.39	0.38	0.72	0.59	0.48	0.40	0.64	0.53	0.47	0.53	0.58	0.34	0.31	0.11	0.82	‐
21	0.34	0.36	0.23	0.37	0.37	0.36	0.68	0.56	0.46	0.38	0.61	0.50	0.45	0.51	0.55	0.32	0.29	0.11	0.77	0.80

## 4 DISCUSSION

This study confirmed a five‐factor structure for the 21‐item SURE, including ‘Drinking and Drug Use’, ‘Self‐ Care’, ‘Relationships’, ‘Material Resources’ and ‘Outlook on Life’ subscales, using binary scoring (1–4, 5, range 21–42) in a sample of people 14 weeks after discharge from Australian RR. The SURE captures personal experiences of recovery [10, 18, 19, 20] and is a useful measure after rescoring to evaluate recovery among people who have completed RR.

IRT analyses and CFA indicated that binary scoring was appropriate in this population and is easily applicable for use in clinical settings. Rescoring the measure as binary accounted for the low response variation for response categories 1–3, which were not resolved by the other proposed scoring options. Analyses within subscales indicated that some items provided more information or information that encompassed other items. SURE development incorporated extensive client and service involvement [10, 17, 18, 19] and consequently includes clinically relevant and meaningful items for multiple stakeholders in the journey of client recovery.

While not all items provide statistical information, all are potentially valuable in evaluating the holistic recovery of clients, helping clients in their self‐reflection, and providing meaningful points for discussion between clients and their clinicians. Hence, all items were retained in the final model aligning with the original aims [9, 10]. The cohort used in this study was post‐treatment, which may have created a ceiling effect. Future research exploring a longer follow‐up period or utilising a timepoint at intake to residential treatment would be helpful. For the overarching Continuing Care Trial, recruitment was closed early due to COVID‐19, which may have influenced recovery for clients discharged from treatment later in the study period. Of the 304 participants in the broader study, 7 were discharged during COVID lockdowns and 77 were in lockdown for part or all of the follow‐up period. Strengths of this study include further validation of the SURE for use following residential rehabilitation in a diverse participant sample with high levels of co‐morbid SUDs, mental health diagnoses and polydrug use, which confirmed the results of the original structure found in the British cohort. The sample was recruited from different treatment services, programs, and geographic regions, and when compared to a broader cohort of clients attending New South Wales non‐government residential rehabilitation [41], no significant differences were found for gender and country of birth, with some differences in age, primary drug of concern and accommodation (Appendix G). Findings are likely generalisable to other residential rehabilitation populations.

The sample involved participants recruited for an intervention trial, some of whom may have engaged in a continuing care intervention. This intervention may have skewed the sample towards more positive recovery outcomes. Considering this, having five responding options may present increased burden on participants completing the PROM, compared to a yes/no response. IRT analyses could only be conducted on three of the five subscales, due to the low number of items within two subscales (Material Resources and Outlook on Life). The Australian social and health care context would likely have influenced participants responses, for example many participants were receiving benefits or financial assistance which may have aided their access to services helping to promote recovery and potentially influence responses on some SURE items (e.g., item 17 ‘I have had a regular income (from benefits, work or other legal sources)’). The final model applied binary, rather than mixed, scoring. This may have resulted in the loss of some relevant statistical information. However, this study sought to strike a balance between statistical rigour, parsimony and ease of use in clinical settings to ensure the measure was fit for purpose.

The item and subscale information curves reflected two identifiable information peaks. This may be representative of two distinct groups in post‐treatment outcomes (higher and lower recovery). Further studies comparing the SURE with binary scoring (1–4,5) post‐RR, with original scoring at treatment entry, are needed to evaluate the presence of higher and lower recovery groups in responding at follow‐up. Using the measure to establish benchmarks post‐residential rehabilitation would help in evaluating change, identifying treatment success, highlighting changes that may indicate relapse, monitoring routine outcomes and evaluating treatments for substance use disorder. The development of quality indicators for the SURE (e.g., change required to demonstrate reliable and clinically significant change) might be useful for researchers and service providers to help understand rates of improvement and deterioration.

The SURE is a valid measure of substance use recovery for assessing outcomes following discharge from RR. The overall findings of this study suggest that the instrument in its original format (21 items, 5‐point Likert scale, 5 factors) can be used for residential clients after discharge, provided responses are rescored to the binary scoring suggested in the study after respondents complete the measure. Rescoring with binary scores provides a parsimonious and efficient method for the use of this measure in populations post discharge from residential rehabilitation. Different scoring standards are recommended for this population compared to clients in other treatment settings or prior to a residential episode. Consistent with the previous study [20], the SURE may not be useful within a given residential rehabilitation episode but has utility for examining post‐discharge recovery.

## AUTHOR CONTRIBUTIONS

Each author certifies that their contribution to this work meets the standards of the International Committee of Medical Journal Editors.

## CONFLICT OF INTEREST STATEMENT

JN was an original author of the development of the SURE. The authors have no additional conflicts of interest to declare. Constraints on publishing: none.

## APPENDIX G

### SAMPLE COMPARISON

Demographic characteristics of substance use recovery evaluator (SURE) participants at baseline (*N* = 225) compared to clients accessing New South Wales non‐goverment organisation residential alcohol and other drug treatment services (*n* = 8161) from Kelly et al. (2021) [41].

**Table:**

	SURE sample (*N* = 225)			NADAbase cohort (*n* = 8161)		
	*N*	%	95% CI	*N*	%	95% CI
Demographic characteristics						
Gender						
Male	132	58.7	[52.23, 65.10]	5276	64.6	[63.61, 65.69]
Female	92	40.9	[34.47, 47.31]	2872	35.2	[34.16, 36.23]
Other/not stated	1	0.4	[0.00, 1.31]	13	0.2	[0.07, 0.25]
Age, years	39.16	10.2	[37.82, 40.50]	35.5	9.9	[35.28, 35.72]
Country of birth						
Australia	202	89.8	[85.82, 93.74]	7372	90.3	[89.69, 90.97]
Other/unknown	23	11.2	[6.26, 14.18]	789	9.7	[9.03, 10.31]
Accommodation						
Rented house or flat	128	56.9	[50.42, 63.36]	4122	50.5	[49.42, 51.59]
Privately owned house or flat	45	20.0	[14.77, 25.23]	1898	23.3	[22.34, 24.17]
No usual residence/homeless	30	13.3	[9.18, 18.48]	523	6.4	[5.88, 6.94]
Other or not known	16	9.8	[3.75, 10.47]	1618	19.8	[18.96, 20.69]
Substance use						
Primary substance of concern^a^						
Amphetamine type substances	88	39.1	[32.73, 45.49]	3124	38.3	[37.23, 39.33]
Alcohol	98	43.6	[37.08, 50.03]	2479	30.4	[29.38, 31.37]
Cannabis	10	4.4	[1.75, 7.14]	798	9.8	[9.13, 10.42]
Benzodiazepines	2	0.9	[0.00, 2.12]	98	1.2	[0.96, 1.44]
Opioids	19	8.5	[4.81, 12.08]	1340	16.4	[15.62, 17.22]
Cocaine	7	3.1	[0.84, 5.38]	130	1.6	[1.32, 1.86]
Other/unknown	1	0.4	[0.00, 1.31]	192	2.4	[2.02, 2.68]

*Note*: Participants in the current study did not significantly differ in gender or country of birth from a larger cohort of clients attending residential treatment in NSW. For accommodation, only proportions of homelessness/no usual accommodation and other accommodations differed. Age, reported as mean (SD), was higher in the current study compared to the larger sample. For primary substance of concern, proportions of amphetamines, benzodiazepines, and cocaine were similar, while alcohol, cannabis, and opioids differed. The difference in primary substance of concern may be due to wording and high proportions of polysubstance use concerns in the current study.

^a^ For the current study, many participants reported polysubstance use as a primary concern. Estimates here show the first reported primary substance of concern.
